# Oxylipin Profiles in Plasma of Patients with Wilson’s Disease

**DOI:** 10.3390/metabo10060222

**Published:** 2020-05-29

**Authors:** Nadezhda V. Azbukina, Alexander V. Lopachev, Dmitry V. Chistyakov, Sergei V. Goriainov, Alina A. Astakhova, Vsevolod V. Poleshuk, Rogneda B. Kazanskaya, Tatiana N. Fedorova, Marina G. Sergeeva

**Affiliations:** 1 Faculty of Bioengineering and Bioinformatics, Moscow Lomonosov State University, Moscow 119234, Russia; ridernadya@gmail.com; 2 Laboratory of Clinical and Experimental neurochemistry, Research Center of Neurology, Moscow 125367, Russia; lopachev@neurology.ru; 3 Belozersky Institute of Physico-Chemical Biology, Lomonosov Moscow State University, Moscow 119992, Russia; alina_astakhova@yahoo.com; 4 SREC PFUR Peoples’ Friendship University of Russia (RUDN University), Moscow 117198, Russia; goryainovs@list.ru; 5 Research Center of Neurology, Moscow 125367, Russia; pol82@yandex.ru; 6 Biological Department, Saint Petersburg State University, Universitetskaya Emb. 7/9, St Petersburg 199034, Russia; magnolia@kwakwa.org

**Keywords:** COX, CYP450, LOX, oxylipins, PUFAs, lipidomics, UPLC-MS/MS, copper, Wilson’s disease

## Abstract

Wilson’s disease (WD) is a rare autosomal recessive metabolic disorder resulting from mutations in the copper-transporting, P-type ATPase gene ATP7B gene, but influences of epigenetics, environment, age, and sex-related factors on the WD phenotype complicate diagnosis and clinical manifestations. Oxylipins, derivatives of omega-3, and omega-6 polyunsaturated fatty acids (PUFAs) are signaling mediators that are deeply involved in innate immunity responses; the regulation of inflammatory responses, including acute and chronic inflammation; and other disturbances related to any system diseases. Therefore, oxylipin profile tests are attractive for the diagnosis of WD. With UPLC-MS/MS lipidomics analysis, we detected 43 oxylipins in the plasma profiles of 39 patients with various clinical manifestations of WD compared with 16 healthy controls (HCs). Analyzing the similarity matrix of oxylipin profiles allowed us to cluster patients into three groups. Analysis of the data by VolcanoPlot and partial least square discriminant analysis (PLS-DA) showed that eight oxylipins and lipids stand for the variance between WD and HCs: eicosapentaenoic acid EPA, oleoylethanolamide OEA, octadecadienoic acids 9-HODE, 9-KODE, 12-hydroxyheptadecatrenoic acid 12-HHT, prostaglandins PGD2, PGE2, and 14,15-dihydroxyeicosatrienoic acids 14,15-DHET. The compounds indicate the involvement of oxidative stress damage, inflammatory processes, and peroxisome proliferator-activated receptor (PPAR) signaling pathways in this disease. The data reveal novel possible therapeutic targets and intervention strategies for treating WD.

## 1. Introduction

Wilson’s disease (WD) is a rare autosomal recessive metabolic disorder resulting from mutations in the copper-transporting, P-type ATPase gene, ATP7B gene, which encodes a copper-transporting P-type ATPase [1]. The enzyme is responsible for the transport of copper into bile from hepatocytes, facilitating its incorporation into apoceruloplasmin to form ceruloplasmin, a major copper-transporting protein in the blood. The mutations lead to copper accumulation in the affected tissues [1,2,3], which causes biochemical deviations, followed by the fluctuating occurrence of hepatic and extrapyramidal symptoms (EPSs), accompanied by the impairment of other organs (for more detail, see recent reviews [4,5,6]). The worldwide prevalence of WD is 1 in 30,000; it is even higher in populations with a high frequency of consanguinity [5]. The manifestations of WD are variable, and, in addition to liver diseases, may include neurological and/or psychiatric symptoms, as well as abnormalities in the blood or kidneys. It is therefore hypothesized that other genetic and/or environmental factors could influence the phenotypes of WD [5,7]. Besides different ATP7B mutations [8], other genetic variations may influence the variability in WD manifestation, such as apolipoprotein E (APOE), human prion protein (PRNP), 5,10-methylenetetrahydrofolate reductase (MTHFR), the interleukin-1 receptor antagonist (IL1RN), peroxisomal catalase, and other genes [7,9]. The influence of epigenetics, environment, age, and sex-related factors on the WD phenotype further complicates diagnosis [3,7,10]. The clear–phenotype correlations for WD are still unclear, although recent epigenetic whole genome screening data may shed light on in-depth pathogenic mechanisms [11]. Obtained from liver and blood samples from patients with WD, the data have shown specific sets of modified genes, enriched for functions in lipid metabolism and inflammatory responses [11]. Such changes can manifest themselves on the level of the organism as a whole in a variety of ways and be the cause of the observed differences in manifestations of the disease. This attracted attention to studying the disease on the level of the metabolome. Understanding the variations in the metabolome may help in identifying variations in the biochemical pathways leading to different manifestations of the disease. High-throughput techniques, such as metabolomic profiling, can deepen our understanding of the disease’s pathogenesis and biology of WD manifestation [12,13], and therefore lead to new therapeutic approaches.

A promising type of metabolomic profiling is oxylipin measurement in the blood or other tissues. Oxylipins, derivatives of omega-3, and omega-6 polyunsaturated fatty acids (PUFAs) are signaling mediators that are deeply involved in innate immunity responses; the regulation of inflammatory responses, including acute and chronic inflammation; and other disturbances related to any system disease [14,15,16,17]. The conversion of PUFAs into oxylipins occurs via three major pathways, named according to their respective key pathway enzymes, such as the cyclooxygenase (COX), lipoxygenase (LOX), and cytochrome P450 monooxygenase (CYP450) branches of metabolism. Besides this, there are non-enzymatic conversions of PUFAs [15]. Due to the diversity of the individual oxylipin functions, it is difficult to predict the general direction of their action. For example, eicosapentaenoic (EPA) and docosahexaenoic (DHA) omega-3 PUFAs, as well as their derivative oxylipins, hydroxyeicosapentaenoic acids (HEPEs) and hydroxydocosahexaenoic acids (HDoHEs), are regarded as anti-inflammatory mediators [14,15]. Arachidonic acid (AA), an omega-6 PUFA, is mainly the source of prostaglandins (PGs), thromboxane (TX), leukotrienes (LTs), and hydroxyeicosatetraenoic acids (HETEs), attributed to groups of proinflammatory oxylipins. Meanwhile, cyclopentenone PGs, non-enzymatic metabolites of PGE2 and PGD2, possess anti-inflammatory features [18]. Oxidative derivatives of α-linolenic acid (ALA) can be transformed into hydroxyoctadecatrienoic (HOTrEs) acids or others [15]. Linoleic acid (LA)-derived oxylipins, such as hydroxyoctadecadienoic (HODEs) acids, agonists of PPARγ [19], or dihydroxyoctadecamonoenoic (DiHOMEs) acids, which are cytotoxic [20], exhibit both pro- and anti-inflammatory features [15]. Taken together, these data show that oxylipin synthesis should not be studied in groups of separate substances, but in terms of oxylipin profiles, which can characterize the different states of the studied organisms.

Indirect data indicate the possibility of oxylipin profile changes in WD. The roles of oxidative stress in the pathogenesis of WD, and dietary omega-3 PUFAs’ usefulness in an animal model of WD suggest that oxylipins may be involved in the clinical manifestation of WD. Moreover, the number of some lipid-related nuclear receptors, such as retinoid X receptor (RXR), peroxisome proliferator-activated receptor α (PPARα), and hepatocyte nuclear factor 4 alpha (HNF4A), is generally decreased in WD animal models and humans [9,21,22,23,24,25]. It is important to note that although oxylipins exhibit multiple effects, they somehow change the state of the innate immunity system [13,14,15]. Oxylipins are important markers of activation of the system, including the regulation of inflammatory resolution processes [13,16]. Despite these facts, the role of the innate immunity system, and oxylipins as parts of the system, is still underestimated in many diseases.

The development of mass spectrometric methods for oxylipin detection made it possible to obtain oxylipin profiles from the plasma of patients with diseases, such as Alzheimer’s disease [24], alcohol-related liver disease [25], or cancer [26]. However, no such studies have been conducted to characterize WD. Therefore, in the present study, UPLC-MS/MS lipidomics analyses were performed to characterize the plasma profiles of patients with various clinical manifestations of WD, compared to healthy subjects in order to identify the oxylipin characteristics of this disease.

## 2. Results

### 2.1. Clinical Characteristics

The study involved 39 WD patients and 16 healthy controls. The anthropometric, demographic, and blood biochemical parameters of the enrolled individuals are presented in Table 1. In total, 25 patients had the akinetic-rigid form, 10 had the trembling form, and 4 had other forms. Biochemical profiling of the blood of WS patients was conducted; data for ceruloplasmin and serum Cu concentrations are shown in Table 1.

### 2.2. Metabolomic Profiling

Using UPLC-MS/MS, we detected a total of 43 metabolites in human plasma (Table S1). Metabolites were from different lipid classes: 3 PUFA (AA, DHA and EPA), 19 AA derivatives, one DGLA derivate, 7 DHA derivatives, 3 EPA derivatives, 7 LA derivatives, and 3 non-PUFA-derived compounds (OEA, AEA, Lyso-PAF).

### 2.3. Volcano Plot Analysis

**Figure 1:**
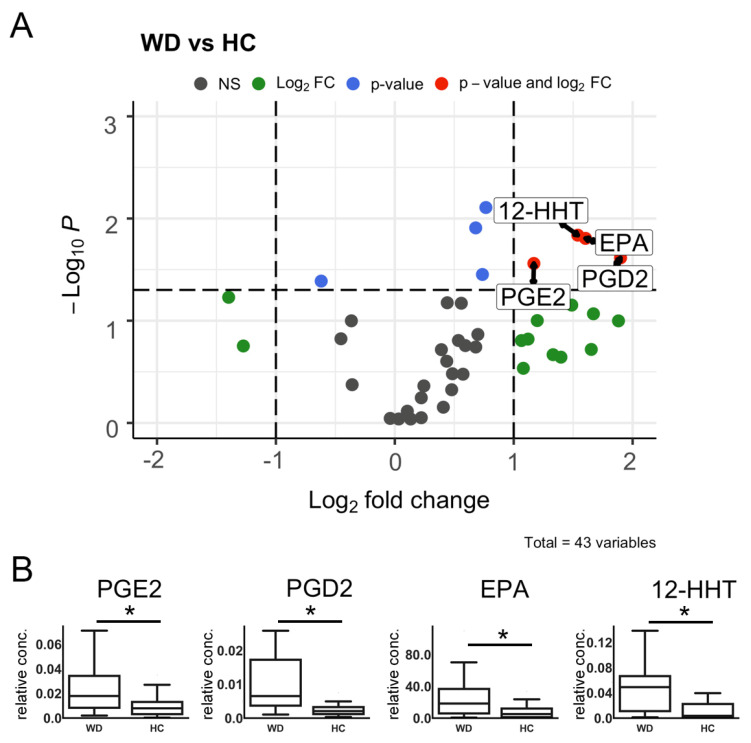
(**A**) Volcano plot indicating significantly changed compounds. The X-axis indicates a log2 fold change of wilson disease WD to HC (healthy control) patients. Y-axis indicates −log10 *p*-values (adjusted). The cut-off for *p*-values is indicated based on Bonferroni correction. Compounds that changed insignificantly are indicated in gray, compounds whose means changed in WD (relative to HCs) more than twofold or less than twofold but insignificantly are indicated in green. Red dots stand for compounds, which changed more than twofold and had a *p*-value (adjusted < 0.05). (**B**) Relative concentrations of separate metabolites that changed significantly in WD patients in comparison with HCs. Pairwise comparison of adjusted means was conducted taking into account the age and sex of patients. * *p* < 0.05 (adjusted for multiple testing).

To evaluate the separate metabolites that differ among WD and HC groups, we performed pairwise comparisons of age and gender-adjusted metabolite concentrations. The results were then illustrated using a volcano plot with Holm–Bonferroni correction (Figure 1). The four metabolites whose concentrations were changed significantly are indicated in red (12-HHT, EPA, PGE2, and PGD2). Barplots of the indicated compounds’ relative concentrations are presented in Figure 1B.

### 2.4. PLS-DA Model

**Figure 2:**
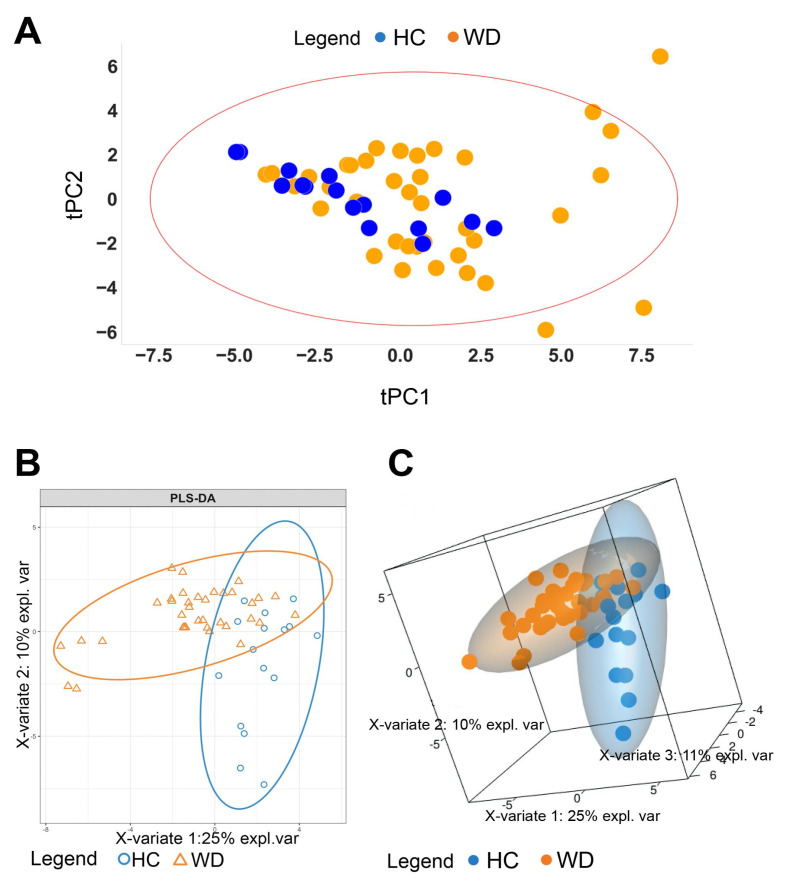
(**A**) The principal component analysis (PCA) performed to verify outliers. The 95% Hotelling T2 confidence interval is indicated as an ellipse. (**B**) The partial least square discriminant analysis (PLS-DA) model discriminating healthy control (HC) and Wilson disease patients (WD). The explained variance of each component is indicated in brackets on the corresponding axis. (**C**) PLS2-DA model represented in 3-D showing separation among the HC and WD patients.

For data analysis, we used normalized concentrations of metabolites (see Section 2.6). The presence of outliers was identified by performing principal component analysis (PCA) to prevent their effects on the model. Hotelling’s T2 test indicated three outliers in the healthy control group. A total of 52 samples, which were placed inside a 95% confidence interval ellipse red bounds (Figure 2A), were used for further analyses. For testing whether WD (Wilson disease) and HC (healthy control) patients could be distinguished based on oxylipin concentrations, the partial least square discriminant analysis (PLS-DA) was performed. The model was evaluated via cross-validation based on the overall error, balanced error rate (BER), and area under curve (AUC) values (Figure S1, Table S2). The optimal number of components was three. Projections on the first two components are presented in Figure 2B, and on the first three components in Figure 2C. Studied groups were separated with a small overlap. For each metabolite, the VIP score was estimated (as described in Section 2.6). The value of this parameter addresses the explained variation between classes in each projection. A total of seven metabolites, including 12-HHT, EPA, 14,15-DHET, 9-HODE, OEA, PGE2, and 9-KODE, with VIP score values > 1.5 are shown in Table 2.

### 2.5. Similarity Matrix

**Figure 3:**
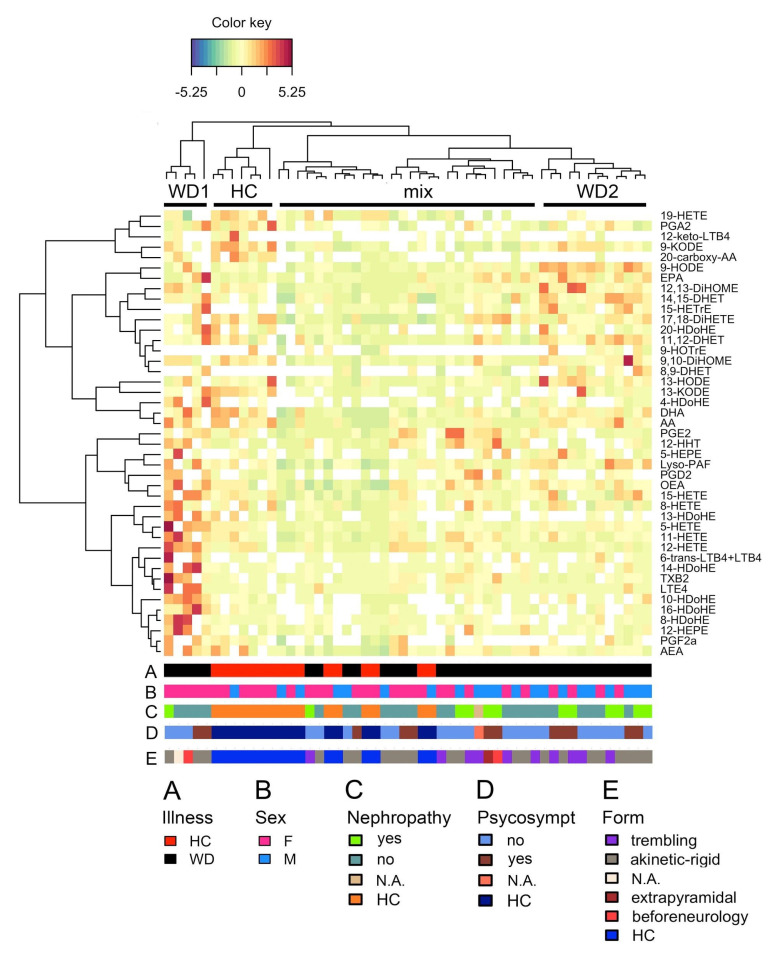
A clustered image map was generated using the Euclidean distance and the complete linkage clustering algorithm. On the figure, each entry of the matrix is colored according to its value; rows represent metabolites, columns represent subjects. Dendrograms are shown on the left side (for patients) and on top (for metabolites). Color bars on the bottom of the picture indicate: (**A**) whether the subjects belongs to the Wilson disease (WD) group or healthy control (HC) group; (**B**) sex distribution: male (M) or female (F); (**C**) nephropathy status: no, yes, HC or data not available (N.A.); (**D**) psychosomatic status: no, yes, HC or N.A.; (**E**) form of the disease: trembling, akinetic-rigid, extrapyramidal, beforeneurology, or HC.

Since oxylipins represent different branches of metabolic pathways [14], we decided to estimate possible interconnections among compounds by calculating the pairwise association matrix, using data obtained using PLS-DA. A clustered image map (CIM), based on a hierarchical clustering of both the rows and the columns, was built using the Euclidean distance and complete linkage clustering algorithm (Figure 3). In the figure, each entry of the matrix is colored according to the association between metabolite concentrations and illness status (X and Y-variables in the model). The red color indicates positive correlation, whereas yellow/green indicates a weaker correlation. Dendrograms are shown on the left side (for metabolites) and on top (for patients). Color bar A indicates whether the patient belongs to WD (black) or HC (red). Based on the dendrogram and illness status, we subdivided the subjects into four groups (bar on the top of heatmap). WD patients can be subdivided into three groups: Not distinguished from healthy donors (mix), with enrichment of HdOHE and HETE compounds, and with DiHETE, DiHOME enrichment (Figure 3).

We further annotated the enrichment of modules using patients’ clinical characteristics. The color bars on the bottom of Figure 3 indicate the clinical and anthropometric annotation of the patients. In total, five bars are presented on the CIM, indicating:

(A) HC/WD patients;
(B) Sex;
(C) Nephropathy status;
(D) Psychosomatic status; and
(E) Form of the disease.

However, all WD patients clustered in a group on the left side turned out to be females (Figure 3). We tested associations with the Cu serum concentration, the severity of motor system dysfunction according to the Shvab scale, age, and the debut age of subjects. There was no clear clustering according to the mentioned parameters (Figure S5A–D).

To test whether there were any differences in separate metabolites between selected groups, we conducted analysis of covariance (ANCOVA) to compare the adjusted means between groups taking into account the variability of the age and sex of patients. To identify which groups were different, pairwise comparisons of the adjusted means with the following Bonferroni multiple testing correction were applied. In the WD1 module patients, 10-HDoHE, 11-HETE, 12-HEPE, 12-HETE, 13-HDoHE, 15-HETE, 16-HDoHE, 5-HETE, and OEA were significantly different from HC (Figure S4). In the WD2 module patients, 10-HDoHE, 9-HODE, and AA were significantly different from HC. The mix module was not different from HCs (Figure S4).

### 2.6. Pathway Enrichment Analysis

**Figure 4:**
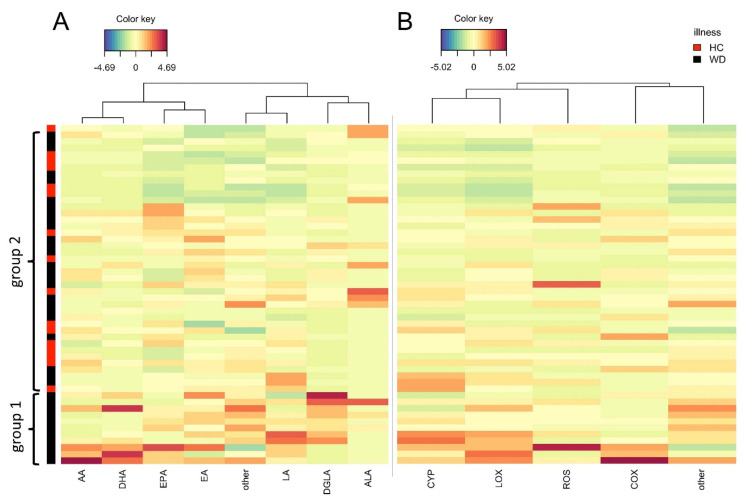
A clustered image map was performed using the Euclidean distance and complete linkage clustering algorithm. All polyunsaturated fatty acids (PUFA) derivatives were summed up according to their (**A**) initial substrate of biochemical pathways or (**B**) pathways’ enzyme origin. In the figure, each entry of the matrix is colored according to its value, rows represent subjects, columns represent metabolites. Dendrograms are shown on the top (for metabolites). The color bar on the left side of the picture indicates whether a subject is WD (black) or a HC (red). Abbreviations: AA: arachidonic acid; DHA: docosahexaenoic acid, EPA: eicosapentaenoic acid; EA: AEA and OEA; LA: linoleic acid; DGLA: dihomo-γ-linolenic acid; ALA: α-linolenic acid; CYP: cytochrome P450 monooxygenase; LOX: lipoxygenase; ROS: reactive oxygen species; COX: cyclooxygenase.

Differences in separate metabolism branches are often a specific trait of biological processes [14,15,16]. This is why after independent analysis of the compounds, we took a step forward and investigated oxylipins as groups. Concentrations of compounds were summed according to their acid precursors (AA, DHA, EPA, ALA, DGLA, EA, EPA) (Figure 4A) or via the metabolic pathways they were derived from (cyclooxygenase (COX), cytochrome P450 monooxygenase (CYP), lipoxygenase (LOX), or non-enzymatic reactive oxygen species (ROS) (Figure 4B). It should be mentioned that in both cases, only the derivatives were summed up; free acids were grouped into the “others” unit. The classification used was in accordance with [15]. Then, a similarity matrix was calculated, and a complete linkage algorithm was performed for the acid precursor matrix (Figure 4A). To simplify the analysis between acid precursor and enzyme pathways, the second CIM was plotted using the order of the corresponding row as in Figure 4A, and clustering was performed only in columns (Figure 4B).

The size of the cluster described in Section 2.5 increased. It was enriched with AA, DHA, EPA, LA, and DGLA metabolites, which indicates respective changes of their concentrations. It should be noted that the correlation between changes in the amount of metabolites of the CYP and LOX pathways was greater than that between their changes and changes in COX metabolites. However, this clustering was not explained by the clinical and demographic parameters mentioned in Section 2.4 and the reason for this standing apart is still unclear. Patients could be subdivided into two clusters independently of the grouping strategy (acids or enzymes), which is similar to the results presented in Figure 3. The bottom cluster of patients was associated with an overall upregulated content of oxylipins. On the other hand, the second cluster of patients did not show such an association.

After analysis of the grouped CIM, we determined a subgroup of patients as group 1. Patients from that group had significantly different concentrations of LOX metabolites (speaking about enzymatic pathways) and significant changes in the concentrations of AA, DGLA, and DHA derivatives and free fatty acids (Figure S5).

## 3. Discussion

Although WD is an autosomal recessive metabolic disorder, it possesses uncertain phenotype–genotype correlations and variability in its clinical manifestations. Understanding the biology of WD pathogenesis and improving diagnostic methods is an urgent problem posed before the science community [27]. Recently developed methods make possible quantitative measurement and analysis of a large number of markers, which, taken together, make up profiles. Oxylipin profiles are unique in that they reflect the activity level of a variety of biochemical processes in the organism and participate in the regulation of various signaling cascades. They possess a characteristic “fingerprint” when certain changes occur, and reflect dynamic characteristics of the organism, which is why interest in oxylipin profiles continues to grow.

Oxylipin profiles were investigated for the study of mechanisms, and as diagnostic markers for diseases, such as Alzheimer’s disease [24], female breast cancer [26], alcohol-related liver disease [25], atherosclerotic diseases [28], and coronary artery disease [29]. Importantly, every disease was characterized by a special set of oxylipins: 9-HODE [26], 20-HETE [25], 8-HETE, LTB4, 9-HODE and 13-HODE [28], 9-HETE, and F(2)-isoprostanes [29]. Although t-test statistics of the oxylipin profiles of 39 (22 female and 17 male) WD patients and 16 (11 female and 5 male) donors allowed four substances (12-HHT, EPA, PGE2, and PGD2) to be revealed, which differ in WD vs HD, it is still not possible to suggest these substances as diagnostic markers, and further investigations are required. However, importantly, our data add new information concerning the biology of WD pathogenesis.

Indeed, analysis of data by VolcanoPlot showed that the patient vs. healthy groups differed significantly across three lipids. PLS-DA analysis revealed five more lipids that explained the difference in oxylipin profiles among WD and HC. Among them, two acids (EPA, OEA); two metabolites of LA, 9-HODE and 9-KODE, which can be attributed to LOX or non-enzymatic branches of metabolism; three metabolites of AA (12-HHT, PGD2, PGE2), attributed to the COX branch; and one AA metabolite from CYP branches of metabolism (14,15-DHET) were included. Although oxylipins possess multiple effects, and the same compound can be traced through various processes [14,15], the following processes can be characterized by the respective compounds: Oxidative stress (9-HODE, 9-KODE, ОЕА, ЕРА), inflammatory markers (9-HODE, 9-KODE, PGE2, 12-ННТ, PGD2), and peroxisome proliferator-activated receptor (PPAR) agonists (9-HODE, 9-KODE, OEA, EPA, 14,15-DHET). The data allow us to make assumptions about the possible signaling pathways involved in this pathology.

The ability of free Cu ions to participate in the formation of reactive oxygen species (ROS) and induce cellular toxicity is known [30]. In the presence of reducing agents (e.g., the superoxide anion radical), Cu2+ can be reduced to Cu+, which catalyzes the formation of hydroxyl radicals from hydrogen peroxide via the Haber–Weiss and Fenton reaction [31]. Therefore, the role of oxidative stress in the pathogenesis of WD is currently under investigation, and peroxisome impairment is suggested to be involved in WD pathophysiology [9,30,31]. Oxidized LA metabolites (HODE/KODE) are traditionally classified as oxidative stress markers [32]. Note that 9-HODE was also marketed as the most upregulated oxylipin species in the plasma of breast cancer patients, which indicates the possibility of oxidative stress involvement in this disease [26]. An interesting finding of our work is an increase of OEA and EPA, substances that are known preventers of oxidative stress [33,34]. Although oxidative stress can be viewed as a common disruption in various pathologies, which may suggest similarities in the oxidized forms of lipids, the variations in the oxylipin profiles obtained in various diseases do not support this point of view [24,25,26,28,29]. Our data sheds light onto some appropriate compensatory mechanisms that may be involved in WD.

An increase in 12-HHT, PGE2, and PGD2 points to the involvement of inflammatory processes in the pathogenesis of WD. This is in accordance with the data obtained in the animal model of WD (the Long-Evans Cinnamon rats), which is characterized by an increase in COX expression, the main enzyme in the synthesis of these substances [35]. Interestingly, dietary omega-3 PUFAs suppress acute hepatitis, prolong the survival of rats, and seem to lead to a decrease in COX expression [35]. Our data, showing an increase in 12-HHT, PGE2, and PGD2, are in accordance with this observation, because it is known that these omega-6 derivatives are inflammatory markers, which may be decreased by supplementation with dietary omega-3 PUFAs [36].

Importantly, the elevation of some oxylipins may lead to the activation of PPARs [37]. Three subtypes of PPAR (PPARα, PPARβ, PPARγ) are active regulators at the lipid metabolism and inflammation crossroad [38]. Recent studies have shown that PPARα and PPARγ are associated with steatosis and impairment of the antioxidant system in the liver of WD patients [39]. Inconsistent with this finding, a transcriptome analysis of the liver in the mouse model of Wilson’s disease under copper-transporting, P-type ATPase gene Atp7b knockout identified the PPAR signaling pathway as a high-copper-responsive target pathway [40].

It is noteworthy that while PPARγ increased, PPARα mRNA expression is decreased with increased severity of WD [41]. This may reveal the “PPAR triad” mechanism that was conjectured for other cells, which respond to an excess of various types of PPAR ligands [42]. There are few data concerning the mechanisms of different PPAR-type changes in the presence of their ligands’ excess at the organism level. Our data suggest that the increased number of PPAR ligands in the blood of WD patients may be associated with some kind of regulatory compensatory mechanism associated with the PPARs system. Our data also single out this signaling pathway for further consideration as being involved in the clinical manifestations of WD. Indeed, among the investigated substances, PPARα agonists were determined by OEA [41], EPA [37], and 14,15-DHET [43]. 9-HODE is an endogenous activator and ligand of PPARγ [19]. In this context, the question remains regarding the role of PGD2 and PGE2. Besides action via specific G-protein-coupled receptors, these prostaglandins are converted in the course of inflammatory reactions into prostaglandins 15d-PGJ2 and PGA2, respectively. Compounds with anti-inflammatory properties are formed, which activate PPARα and PPARγ [18]. At present, the question remains open whether an increase in PPAR agonists in the blood plasma of patients with WD is a protective mechanism that weakens the severity of the clinical course of the disease or, conversely, aggravates the symptoms. We were not able to find data on the use of synthetic agonists of PPAR in WD models. It is likely that fibrates and thiazolidinediones may be used as potential therapeutic agents for WD. Further research is required to elucidate the molecular mechanisms by which PPAR agonists may exert their effects in WD pathogenesis.

Beside PPARs, we cannot exclude oxylipins’ involvement in the regulation of the activity of other nuclear receptors, because it is known that lipid-related nuclear receptors change in WD patients or animal models [22,23]. Decreased binding of the nuclear receptors FXR, RXR, HNF4α, and LRH-1 to promoter response elements and decreased mRNA expression of nuclear receptor target genes [22] and dysregulation of LXR/RXR heterodimers [23] may also be compensated by increased lipid agonist concentrations. Our data are consistent with these results, but further research is required to understand the mechanisms.

An important finding of our work is the subdivision of the patients into groups relative to the oxylipin profiles. Patients from the group had significantly different concentrations of LOX metabolites and had significant changes in the concentration of AA, DGLA, and DHA derivatives and free fatty acids. It is not yet clear which parameter underlies this division, since we did not find any correlation with gender or age. The subdivision into groups can reflect the various states of the innate immunity system. Along with various cytokines, oxylipins are part of the innate immunity system, being proinflammatory substances, as well as mediators of resolution [13]. Innate immune traits are more affected by the environment [44]. Environmental factors, including diet, exercise, stress, and toxins, profoundly impact the phenotypes of diseases, and WD is among them [7]. It is worthwhile to assume that the observed separation of patients by the oxylipin profile reflects a phenotypic response to environmental factors and therefore the current state of the innate immune system. This aspect requires further investigation of the metabolites that we found to be characteristic of WD.

## 4. Materials and Methods 

### 4.1. Reagents

The oxylipins standards were as follows: 6-keto PGF1α-d4 (cat.no. 315210), TXB2-d4 (cat.no. 319030), PGF2α-d4 (cat.no. 316010), PGE2-d4 (cat.no. 314010), PGD2-d4 (cat.no. 312010), leukotriene (LT) C4-d5 (cat.no. 10006198), LTB4-d4 (cat.no. 320110), 5(S)-HETE-d8 (cat.no. 334230), 12(S)-HETE-d8 (cat.no. 334570), 15(S)-HETE-d8 (cat.no. 334720), oleoyl ethanolamide-d4 (cat.no. 9000552), EPA-d5 (cat.no, 10005056), DHA-d5 (cat.no. 10005057), and AA-d8 (cat. No. 390010) (Cayman Chemical, Ann Arbor, MI, USA). An Oasis^®^ PRIME HLB solid-phase lipid extraction cartridge (60 mg, 3 cc, cat.no. 186008056) was obtained from Waters, Eschborn, Germany.

### 4.2. Population and Study Design

This was an observational study with 55 recruited people: 39 patients with WD and 16 healthy controls. In total, 39 individuals with Wilson’s disease admitted to the regular inpatient treatment in the Research Center of Neurology (Moscow, Russia) were recruited for the study. Inclusion criteria for the WD patients included the following clinical and laboratory signs of the disease: Debut of the illness in childhood, adolescence, or adulthood (most often up to 35 years old); combined brain and internal organ damage (liver cirrhosis, hepatolienal syndrome, portal hypertension, tubular nephritis, etc.); damage to the central nervous system in the form of extrapyramidal syndrome; and systemic discuprinosis with impaired copper-ligand metabolism.

WD exclusion criteria included the following: Disease manifestation after 35 years; autosomal dominant type of inheritance; the presence of anamnestic, clinical, or paraclinical signs of another disease that can cause similar symptoms; hallucinations not related to medication; the presence of dementia or signs of impaired cortical function (aphasia, apraxia, etc.); the slowing down of vertical saccades or vertical gaze paralysis; a positive history of inflammatory diseases; chronic diseases and metabolic disorders; treatment with nonsteroidal anti-inflammatory drugs (NSAIDs) or corticosteroids during the last month; and pregnancy or breast-feeding during the study visit.

In total, 16 healthy individuals not affected by neurodegenerative disorders as verified by clinical examination were included in the study. They were recruited among people undergoing periodic health examinations at the same center. The exclusion criteria for healthy controls were the same as for patients with WD.

The Ethics Committee of the Research Center of Neurology approved this study (protocol №4-4/19 15.05.19), and informed written consent was obtained from each patient and control according to the guidelines approved under this protocol (Article 20, Federal Law “Protection of Health Right of Citizens of Russian Federation” N323- FZ, 11.21.2011).

### 4.3. Clinical Evaluation

The criteria for inclusion in the group of patients with WD consisted of clinical and laboratory signs of the disease: Debut in childhood, adolescence, and adulthood (most often up to 35 years old); combined damage to the brain and internal organs (cirrhosis, hepatolienal syndrome, portal hypertension, tubular nephritis, etc.); central nervous system (CNS) damage in the form of extrapyramidal syndrome, including tremor, stiffness, dysarthria, dysphagia, cognitive impairment, and dysphoria; and extra-neural symptoms, including hepatosplenomegaly, hemorrhages, failure of levodopa treatment (prescribed in connection with Parkinson’s syndrome). Criteria for laboratory diagnosis of WD: Kaiser–Fleischer corneal ring (when using a slit lamp); a decrease in the concentration of ceruloplasmin copper ligand protein in the blood serum; hypersecretion of copper with urine; increase in the concentration of free copper in blood serum; a decrease in the concentration of total copper in serum; decreased serum zinc concentration; increased copper concentration in liver biopsy specimens; DNA diagnostics (detection of mutations in the ATP7B gene); and high therapeutic effect when using copper eliminating chelates (D penicillamine, trientin) and zinc preparations. An additional diagnostic criterion for WD was the result of neuroimaging (computed tomography and magnetic resonance imaging (CT, MRI)), showing an atrophic process in the cerebral hemispheres, cerebellum, subcortical structures with a corresponding expansion of subarachnoid spaces, and the ventricular system, as well as foci in the area of lenticular nuclei, globus pallidus, and visual hillock [26,27].

### 4.4. Blood Sample Collection

Taking into account the reported serum oxylipin variety during daytime [45], all blood sample collection was conducted in the morning in the fasted state. The plasma was obtained immediately after blood sampling, aliquoted, and stored at −80 °C for further analysis.

### 4.5. UPLC-MS/MS Conditions and Sample Preparation

Samples were prepared for MS analysis by the solid-phase extraction (SPE) method using an Oasis^®^PRIME HLB cartridge (60 mg, 3 cc). For eicosanoid extraction, plasma (900 μL) was deproteinized with 1 mL of methanol, vortexed, and centrifuged at 12,000 rpm for 5 min at ambient temperature. The supernatant was diluted 1:6 with mQ water containing 0.1% formic acid for the next steps of SPE. Then, the sample was loaded, and the cartridge was washed with 2 mL of 15% methanol containing 0.1% formic acid, after which the lipids were sequentially eluted with 500 μL of anhydrous methanol and 500 μL of acetonitrile. The resulting samples were concentrated by evaporation of the solvent under a gentle stream of nitrogen and stored at −80 °C. For the identification of lipid mediators, the respective lipid extracts were analyzed using an 8040 series UPLC-MS/MS mass spectrometer (Shimadzu, Japan) in multiple-reaction monitoring mode at a unit mass resolution for both the precursor and product ions [46]. The selected molecular ions were fragmentized in the gas phase by collision-induced dissociation and analyzed by tandem (MS/MS) mass spectrometry. The studied metabolites were identified and quantified according to the comparison of their multiple reaction monitoring parameters, retention times, and peak areas with the parameters obtained for deuterated internal standard compounds of the same classes (6-keto PGF1α-d4, TXB2-d4, PGF2α-d4, PGE2-d4, PGD2-d4, leukotriene (LT) C4-d5, LTB4-d4, 5(S)-HETE-d8, 12(S)-HETE-d8, 15(S)-HETE-d8, oleoyl ethanolamide-d4, EPA-d5, DHA-d5, AA-d8) (Table S1) using a commercial software method package Lipid Mediator Version 2 (Shimadzu, Tokyo, Japan) according to the manufacturer’s instructions.

Prior to analysis, the plasma oxylipin detection method was validated according to food and drug administration (FDA) recommendations [47]. The stock ethanol solution containing 2 ng of each of the 15 deuterated oxylipin standards was prepared. Calibration samples (1.4, 1, 0.4, 0.2, and 0 ng/probe) were obtained by further dilution of the stock solution containing the same total volume of plasma. For each standard intraday, the interday reproducibility and relative standard deviation (RSD, %) were determined. The accuracy was measured with spiked standards for 3 concentration ranges: 0.2–0.8 ng/probe, 0.9–1.3 ng/probe, and 1.4–2 ng/probe. The limit of detection (LOD) was determined as the signal to noise ratio = 3, and the limit of quantification (LOQ) as the signal to noise ratio = 3. Signal to noise ratio was calculated as the standard error of the regression/slope. Results are presented in Table S2.

### 4.6. Experimental Data Analysis and Statistics

Comparison of the relative concentrations was performed using the two-sample two-sided *t*-test, followed by Bonferroni–Holm correction for multiple comparisons. *p* < 0.05 was considered as statistically significant.

Metabolomics data was analyzed using the mixOmics R package version 6.1.1 [48]. After data normalization on internal standards, peak area mean centering and unit variance scaling was applied. Class separation was analyzed by partial least square discriminant analysis (PLS-DA). The quality of the built model was estimated using leave-one-out cross validation. Each round of cross-validation included training on the bigger data subset and validation on the randomly selected sample. The model’s predictive performance was estimated based on the validation results combined over rounds. This procedure was repeated for different numbers of components in the PLS-DA model. The overall error, balanced error rate, and AUC were obtained for each number of components (Figures S1 and S2, Table S5).

After building the PLS-DA model with 3 components, VIP scores for each investigated metabolite were calculated. A VIP score is a weighted sum of squares of the PLS loadings regarding the explained variation in each projection. A cutoff for VIP-scores was accepted as 1.5 according to the metabolomics standard initiative (level MSI = 1).

Analysis of covariance (ANCOVA) was used to compare the means of single metabolites between studied groups, taking into account sex and age. ANCOVA was performed using rstatix package for R. Pairwise comparisons of relative metabolites’ concentrations was performed using function emmeans_test (rstatix package), also taking into consideration age and sex as covariates. Analysis was followed by Bonferroni–Holm correction for multiple comparisons. *p* < 0.05 was considered as statistically significant.

## 5. Conclusions

In conclusion, our findings reveal alterations of the plasma oxylipin profiles in Wilson’s disease patients, and heterogeneity in patients relative to the oxylipin profiles. Eight lipids were found to vary between HC and WD: EPA, OEA, 9-HODE, 9-KODE, 12-HHT, PGD2, PGE2, and 14,15-DHET; among them, PGE2, PGD2, 12-HHT, and EPA changed significantly (based on the pairwise comparison of means adjusted for sex and age).

The biological significance of these compounds indicates the involvement of oxidative stress damage, inflammatory processes, and PPAR signaling pathways in this disease. The data reveal novel possible therapeutic targets and intervention strategies for treating WD.

## Figures and Tables

**Table 1:** Demographic parameters of the patients, disease characteristics, and medications.

**Wilson Disease Patients**						
Sex	F (n = 22)			M (n = 17)		
	mean	sd	n	mean	sd	n
Age	35.68	13.17		32.18	12.36	
Serum Cu, mkM	8.5	4.3		9.62	4.36	
Shvab scale, %	80.95	18.41		67.5	22.36	
Leipzig score	7.32	2.19		6.42	2.57	
Ceruloplasmin, mg/dL	9.61	7.2		12.78	8.02	
Height, cm	169.95	5.55		180.8	7.44	
Longevity illness, years	13.86	11.34		9.81	9.32	
Longevity treatment, years	12.86	11.25		8.21	8.75	
Weight, kg	61.2	13.27		75.67	12.21	
Form (akinetic-rigid/trembling/others)			2014/6/2			2011/4/2
Nephropathy			5			8
Portal hypertension			5			11
Psycoproductive somatic			8			6
**Healthy Donors**						
	F (11)			M (5)		
	mean	sd	n	mean	sd	n
Age	37.88	15.96		49.2	12.19	

**Table 2:** Variable importance in projection (VIP) scores are shown for 7 metabolites. A cutoff value of 1.5 is established for VIP selection.

Name	12-HHT *	EPA *	14,15-DHET	9-HODE	OEA	PGE2 *	9-KODE
VIP-scores	1.899456	1.741633	1.739218	1.624940	1.617023	1.594828	1.837164

* volcano plot indicating significantly changed compounds, *p* < 0.05 (adjusted for multiple testing).

## Supplementary Materials

The following are available online at https://www.mdpi.com/2218-1989/10/6/222/s1, Figure S1: The Balanced Error Rate (BER) and overall error rate estimated via cross-validation for different component numbers, Figure S2: ROC (receiver operating characteristic) curve for chosen number of components, Figure S3: A clustered image map was generated using euclidean distance and complete linkage clustering algorithm, Figure S4: Relative concentrations of separate metabolites which changed significantly in WD1 and/or WD2 in comparison with HC, Figure S5: Relative concentrations of summed metabolites which changed significantly between group1 and group2, Table S1: UPLC-MS/MS parameters of the identified lipids, Table S2: Intraday reproducibility, Table S3: Interday reproducibility, Table S4: Accuracy, LOD, LOQ, Table S5: AUC (Area Under Curve) values and p-value estimated for different number of components, Table S6: Mean +/− standard deviation of relative concentrations for healthy controls (HC) and Wilson disease patients (WD), Table S7: Source acid and metabolic enzyme for the analyzed oxylipins.

## Abbreviations

AA	Arachidonic acid
COX	Cyclooxygenase
CYP450	Cytochrome P450 monooxygenase
DHA	Docosahexaenoic acid
DiHOME	Dihydroxyoctadecamonoenoic acid
HC	Healthy control
HDoHE	Hydroxydocosahexaenoic acid
HETE	Hydroxyeicosatetraenoic acid
HODE	Hydroxyoctadecadienoic acid
LA	Linoleic acid
LOX	Lipoxygenase
PG	Prostaglandin
PUFAs	Polyunsaturated fatty acids
WD	Wilson disease
UPLC-MS/MS	Ultra-performance liquid chromatography-tandem mass spectrometry
