# Supplementary material for: Oxylipin Profiles in Plasma of Patients with Wilson’s Disease
Source: Metabolites. 2020 May 29;10(6):222. doi: 10.3390/metabo10060222 (PMC7345781; doi:10.3390/metabo10060222)
Supplement: Supplementary file 1 [file metabolites-10-00222-s001.pdf]

SUPPLEMENTARY

Oxylipin profiles in plasma of patients with Wilson's disease

Nadezhda V. Azbukina[1] Alexander V. Lopachev[2], Dmitry V. Chistyakov[3*], Sergei V. Goriainov[4], Alina A. Astakhova[3], Vsevolod V. Poleshuk[2], Rogneda B. Kazanskaya[5], Tatiana N. Fedorova[2*], Marina G. Sergeeva[3*]

[1]Faculty of Bioengineering and Bioinformatics, Moscow Lomonosov State University, 119234 Moscow, Russia;

[2]Laboratory of Clinical and Experimental neurochemistry, Research Center of Neurology, 125367, Moscow, Russia;

[3]Belozersky Institute of Physico-Chemical Biology, Lomonosov Moscow State University, Moscow 119992, Russia;

[4]Peoples' Friendship University of Russia, Moscow, 117198 Russia;

5Biological Department, Saint Petersburg State University, Universitetskaya Emb. 7/9, 199034, St Petersburg, Russia.

***Corresponding authors:**

Dmitry V. Chistyakov and Marina G. Sergeeva, Belozersky Institute of Physico-Chemical Biology, Lomonosov Moscow State University,1-40 Leninskye Gory, Moscow, 119992 Russia; Tatiana N. Fedorova, Laboratory of Clinical and Experimental neurochemistry, Research Center of Neurology, Volokolamskoe shosse, 80, Moscow, Russian Federation, 125367.

Table S1. UPLC-MS/MS parameters of the identified lipids.

Compound name	CAS Number	Retention time (min)	Target ion (m/z)		+/-	Matching internal standard
TXB2	54397-85-2	9,465	369,2	195,1	(−)	TXB2-d4
PGF2a	551-11-1	10,27	353,2	193,1	(−)	PGF2a-d4
PGE2	363-24-6	10,7	351,2	271,2	(−)	PGE2-d4
PGD2	41598-07-6	11,1	351,2	271,2	(−)	PGD2-d4
LTE4	75715-89-8	12,72	440,3	189,1	(+)	LTC4-d5
PGA2	13345-50-1	13,02	333,2	271,2	(−)	PGD2-d4
17,18-DiHETE	116477-53-3	13,94	335,2	247,2	(−)	LTB4-d4
LTB4	71160-24-2	14,12	335,2	195,1	(−)	LTB4-d4
12,13-DiHOME	263399-35-5	14,46	313,2	183,1	(−)	LTB4-d4
9,10-DiHOME	263399-34-4	14,63	313,2	201,2	(−)	LTB4-d4
12-keto-LTB4	136696-10-1	14,74	333,2	179,1	(−)	LTB4-d4
14,15-DHET	77667-09-5	15,04	337,2	207,2	(−)	LTB4-d4

12-HHT	54397-84-1	15,155	279,2	179,1	(−)	LTB4-d4
11,12-DHET	192461-95-3	15,36	337,2	167,1	(−)	LTB4-d4
8,9-DHET	192461-96-4	15,54	337,2	127,1	(−)	LTB4-d4
9-HOTrE	89886-42-0	15,66	293,2	171,1	(−)	LTB4-d4
20-carboxy-AA	79551-84-1	15,67	333,2	297,2	(−)	LTB4-d4
19-HETE	115461-40-0	15,95	319,2	275,2	(−)	LTB4-d4
12-HEPE	81187-21-5	16,385	317,2	179,1	(−)	15-HETE-d8
5-HEPE	83952-40-3	16,45	317,2	115,1	(−)	15-HETE-d8
Lyso-PAF	52691-62-0	16,49	482,3	104,2	(+)	PAF-d4
13-HODE	73804-64-5	16,6	295,2	195,1	(−)	15-HETE-d8
9-HODE	98524-19-7	16,6	295,2	171,1	(−)	15-HETE-d8
20-HDoHE	90906-41-5	16,77	343,2	241,2	(−)	15-HETE-d8
15-HETE	73836-87-0	16,93	319,2	219,2	(−)	15-HETE-d8
13-KODE	54739-30-9	17	293,2	113,1	(−)	15-HETE-d8
16-HDoHE	90780-51-1	17,05	343,2	233,2	(−)	12-HETE-d8
9-KODE	54232-59-6	17,12	293,2	185,1	(−)	12-HETE-d8
11-HETE	73804-65-6	17,125	319,2	167,1	(−)	12-HETE-d8
13-HDoHE	90780-53-3	17,15	343,2	193,1	(−)	12-HETE-d8
10-HDoHE	90780-50-0	17,2	343,2	153,1	(−)	12-HETE-d8
8-HETE	79495-84-4	17,2	319,2	155,1	(−)	12-HETE-d8
14-HDoHE	87042-40-8	17,24	343,2	205,2	(−)	12-HETE-d8
12-HETE	54397-83-0	17,28	319,2	179,1	(−)	12-HETE-d8
8-HDoHE	90780-54-4	17,37	343,2	109,1	(−)	5-HETE-d8
5-HETE	70608-72-9	17,38	319,2	115,1	(−)	5-HETE-d8
15-HETrE	92693-02-2	17,44	321,2	221,2	(−)	5-HETE-d8
4-HDoHE	90906-40-4	17,67	343,2	101,1	(−)	5-HETE-d8
AEA	94421-68-8	18,21	348,2	62,1	(+)	OEA-d4
OEA	111-58-0	19,14	326,2	62,1	(+)	OEA-d4
EPA	10417-94-4	19,415	301,2	257,2	(−)	EPA-d5
DHA	6217-54-5	20,16	327,2	283,2	(−)	DHA-d5
AA	506-32-1	20,32	303,2	303,2	(−)	AA-d8

Table S2. Intraday reproducibility

	**Slope**	**Intercept**	**Error (σ) slope**	**Error (σ) intercept**	**Relative error slope**	**Relative error intercept**	**R^2**	**Standard error of regression (SER)**	**Relative standard deviation (RSD. %)**
**6-keto-PGF1a-d4**	2444.071	-38.804	82.861	85.872	0.034	-2.213	0.973	311.855	6.191
**TXB2-d4**	7593.666	178.751	297.693	308.511	0.039	1.726	0.962	1120.394	7.015

**PGF2a-d4**	1948.939	42.945	126.526	131.124	0.065	3.053	0.903	476.192	11.638
**PGE2-d4**	6875.689	246.933	319.630	331.246	0.046	1.341	0.947	1202.958	8.259
**PGD2-d4**	5306.940	8.195	176.488	182.902	0.033	22.319	0.973	664.229	6.026
**LTC4-d5**	2997.273	67.945	127.417	132.047	0.043	1.943	0.956	479.546	7.612
**PGA2-d4**	8550.508	312.019	304.138	315.190	0.036	1.010	0.968	1144.651	6.304
**LTB4-d4**	3066.559	-6.019	140.428	145.531	0.046	-24.177	0.951	528.512	8.312
**15-HETE-d8**	3344.472	63.796	201.106	208.414	0.060	3.267	0.916	756.880	10.796
**12-HETE-d8**	1661.877	-52.008	126.950	131.563	0.076	-2.530	0.877	477.786	14.061
**5-HETE-d8**	2753.773	44.770	148.207	153.593	0.054	3.431	0.932	557.792	9.677
**OEA-d4**	123070.914	21076.465	7207.485	7469.403	0.059	0.354	0.907	27126.025	9.396
**EPA-d5**	968.423	4.449	45.248	31.218	0.047	7.016	0.952	174.788	8.485
**DHA-d5**	583.910	11.467	41.140	42.635	0.070	3.718	0.888	154.835	12.647
**15-deoxyPGJ2-d4**	7493.587	685.609	525.081	544.162	0.070	0.794	0.882	1976.190	12.056

Table S3. Interday reproducibility

	**Slope**	**Intercept**	**Error (σ) slope**	**Error (σ) intercept**	**Relative error slope**	**Relative error intercept**	**R^2**	**Standard error of regression (SER)**	**Relative standard deviation (RSD. %)**
**6-keto-PGF1a-d4**	3306.820	-760.694	350.301	476.203	0.106	-0.626	0.898	526.787	11.147
**TXB2-d4**	10491.141	-1193.387	1569.479	2133.569	0.150	-1.788	0.832	2216.397	13.322
**PGF2a-d4**	2570.495	421.726	687.243	934.247	0.267	2.215	0.663	960.557	19.150
**PGE2-d4**	6875.689	246.933	319.630	331.246	0.046	1.341	0.947	1202.958	8.259
**PGD2-d4**	5253.891	452.015	420.706	469.987	0.080	1.040	0.910	649.210	8.810
**LTC4-d5**	3082.120	90.765	244.881	273.566	0.079	3.014	0.910	410.043	9.217

**PGA2-d4**	12064.274	295.065	2673.015	3633.727	0.222	12.315	0.720	3570.758	16.196
**LTB4-d4**	4370.303	-466.032	781.735	1062.699	0.179	-2.280	0.778	1072.299	14.599
**15-HETE-d8**	3299.651	483.735	417.004	465.851	0.126	0.963	0.805	699.740	13.149
**12-HETE-d8**	2157.892	-137.694	402.466	547.117	0.187	-3.973	0.770	565.802	16.700
**5-HETE-d8**	2668.431	487.706	360.060	402.237	0.135	0.825	0.785	553.064	13.001
**OEA-d4**	102653.248	54762.382	9706.689	10843.723	0.095	0.198	0.890	20271.788	9.002
**EPA-d5**	4453.004	45.328	229.126	202.359	0.051	4.464	0.940	451.668	10.911
**DHA-d5**	677.110	26.284	87.825	110.185	0.130	4.192	0.743	178.435	13.099
**15-deoxyPGJ2-d4**	4669.416	5804.435	1430.199	1944.229	0.306	0.335	0.733	2579.111	16.831

Table S4. Accuracy, LOD, LOQ.

**Other parameters**			**Accuracy**		
	**LOD (3SER/Slope) (ng)**	**LOQ (10SER/Slope) (ng)**	**0.2-0.8 ng/probe**	**0.9-1.3 nh/probe**	**1.4-2 ng/probe**
**6-keto-PGF1a-d4**	0.478	1.593	121.792	74.277	60.848
**TXB2-d4**	0.634	2.113	126.785	84.188	64.595
**PGF2a-d4**	1.121	3.737	121.831	86.374	56.817
**PGE2-d4**	0.525	1.750	130.322	75.973	62.709
**PGD2-d4**	0.371	1.236	126.294	79.850	66.416
**LTC4-d5**	0.399	1.330	128.523	86.228	82.958
**PGA2-d4**	0.888	2.960	125.301	76.412	68.110
**LTB4-d4**	0.736	2.454	111.328	75.837	68.053
**15-HETE-d8**	0.636	2.121	64.111	71.416	59.792
**12-HETE-d8**	0.787	2.622	134.304	61.427	76.821

**5-HETE-d8**	0.622	2.073	90.726	80.260	81.628
**OEA-d4**	0.592	1.975	28.132	58.189	68.068
**EPA-d5**	0.304	1.014	33.678	69.117	46.610
**DHA-d5**	0.791	2.635	13.867	73.099	75.697
**15-deoxyPGJ2-d4**	1.657	5.523	145.978	73.897	60.536

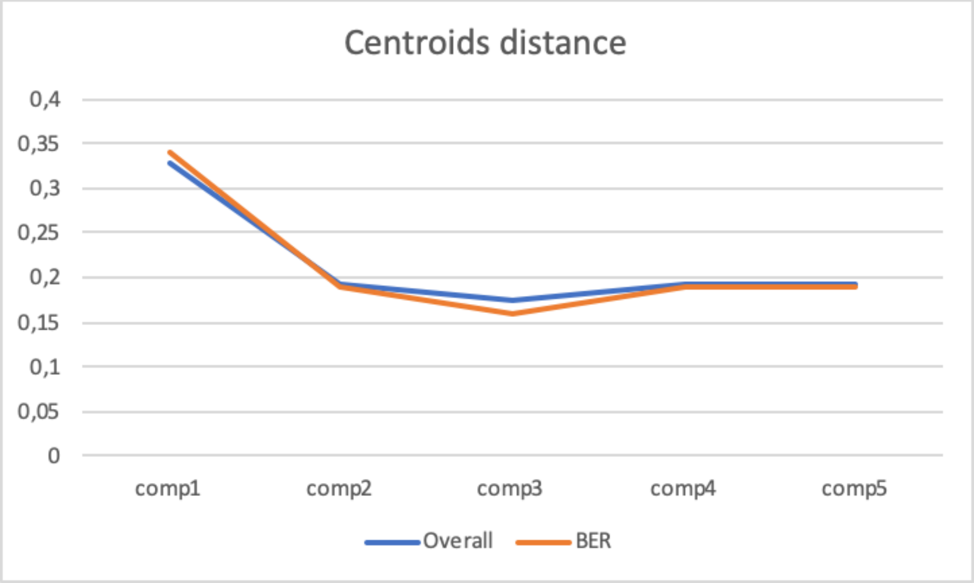

Figure S1. The Balanced Error Rate (BER) and overall error rate estimated via cross-validation for different component numbers.

Table S5. AUC (Area Under Curve) values and p-value estimated for different number of components.

**ncomp**	**AUC**	**p-value**
ncomp1	0.7917000	0.00086591
ncomp2	0.8958	6.172e-06
ncomp3	0.9097	2.883e-06
ncomp4	0.8785	1.545e-05
ncomp5	0.8611	3.725e-05

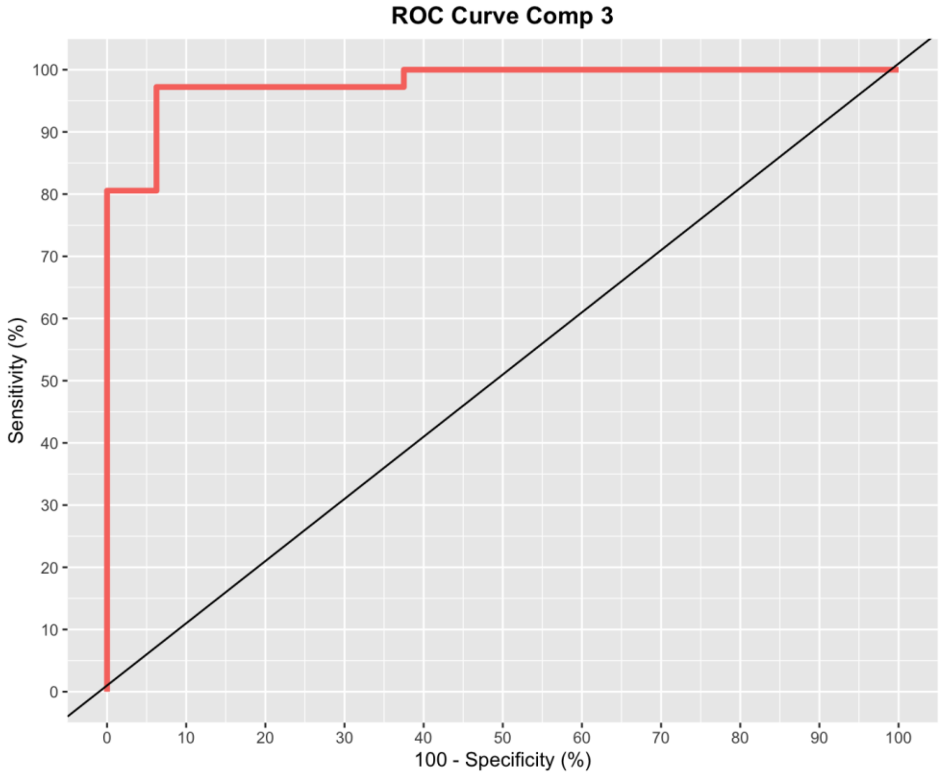

Figure S2. ROC (receiver operating characteristic) curve for chosen number of components (3).

Table S6. Mean +/- standard deviation of relative concentrations for healthy controls (HC) and Wilson disease patients (WD).

	**Relative concentration**	
**illness**	**HC**	**WD**
10-HDoHE	0.0121±0.01	0.0271±0.0274
11,12-DHET	0.1055±0.068	0.1135±0.0692
11-HETE	0.0573±0.045	0.0918±0.0754
12,13-DiHOME	1.1567±0.717	1.6706±1.2151
12-HEPE	0.0131±0.0097	0.0336±0.0422
12-HETE	0.8281±0.7086	1.2521±1.221
12-HHT	0.0118±0.0143	0.0464±0.0372
12-keto-LTB4	0.0433±0.0578	0.0208±0.0139

13-HDoHE	0.0093±0.0112	0.0145±0.0142
13-HODE	1.2115±1.0144	1.3636±0.9827
13-KODE	0.1574±0.1113	0.1131±0.1163
14,15-DHET	0.1221±0.0739	0.1912±0.0864
14-HDoHE	0.0438±0.0267	0.103±0.16
15-HETE	0.0378±0.0253	0.0553±0.0402
15-HETrE	0.0151±0.0081	0.0356±0.0333
16-HDoHE	0.0177±0.0128	0.0317±0.0424
17,18-DiHETE	0.0647±0.0536	0.0763±0.0403
19-HETE	0.907±0.3395	0.7024±0.2777
20-HDoHE	0.0132±0.0136	0.0145±0.0156
20-carboxy-AA	0.1172±0.0994	0.0388±0.0495
4-HDoHE	0.0248±0.0181	0.0207±0.0239
5-HEPE	0.0069±0.0069	0.016±0.0165
5-HETE	0.084±0.0591	0.1237±0.1374
6-trans-LTB4+LTB4	0.0064±0.0039	0.0333±0.0671
8,9-DHET	0.0056±0.0002	0.0111±0.0068
8-HDoHE	0.0197±0.0123	0.0505±0.0827
8-HETE	0.0232±0.0174	0.0272±0.0193
9,10-DiHOME	1.5115±1.067	1.5454±1.4252
9-HODE	0.4867±0.1988	0.8474±0.5325
9-HOTrE	0.0049±0.0049	0.007±0.0027
9-KODE	0.0837±0.0541	0.0542±0.0339
AA	9.5848±7.1545	8.6858±5.5443
AEA	1.8321±1.5384	2.3297±1.1781
DHA	46.2452±36.2903	41.8502±23.264

EPA	8.5929±9.7091	24.6221±23.2701
LTE4	0.0194±0.0204	0.0912±0.1524
Lyso-PAF	326.5978±156.9061	431.2231±197.1076
OEA	10.8608±6.6427	16.7657±8.5828
PGA2	0.0438±0.0251	0.0319±0.0197
PGD2	0.0027±0.0023	0.0095±0.0076
PGE2	0.0102±0.0101	0.0231±0.0186
PGF2a	0.0045±0.0029	0.0066±0.0036
TXB2	0.1087±0.1195	0.4916±0.6527

Table S7. Source acid and metabolic enzyme for the analyzed oxylipins.

**Chemical**	**Acid**	**Enzyme**
10-HDoHE	DHA	LOX
11,12-DHET	AA	CYP
11-HETE	AA	LOX
12,13-DiHOME	LA	CYP
12-HEPE	EPA	LOX
12-HETE	AA	LOX
12-HHT	AA	COX
12-keto-LTB4	AA	LOX
13-HDoHE	DHA	LOX
13-HODE	LA	LOX
13-KODE	LA	LOX
14,15-DHET	AA	CYP
14-HDoHE	DHA	LOX
15-HETE	AA	LOX

15-HETrE	DGLA	LOX
16-HDoHE	DHA	LOX
17,18-DiHETE	EPA	CYP
19-HETE	AA	CYP
20-HDoHE	DHA	CYP
20-carboxy-AA	AA	CYP
4-HDoHE	DHA	LOX
5-HEPE	EPA	ROS
5-HETE	AA	LOX
6-trans-LTB4+LTB4	AA	LOX
8,9-DHET	AA	CYP
8-HDoHE	DHA	LOX
8-HETE	AA	LOX
9,10-DiHOME	LA	CYP
9-HODE	LA	LOX
9-HOTrE	ALA	LOX
9-KODE	LA	LOX
AA	-	-
AEA	EA	-
DHA	-	-
EPA	-	-
LTE4	AA	LOX
Lyso-PAF	-	-
OEA	EA	-
PGA2	AA	COX
PGD2	AA	COX

PGE2	AA	COX
PGF2a	AA	COX
TXB2	AA	COX

A

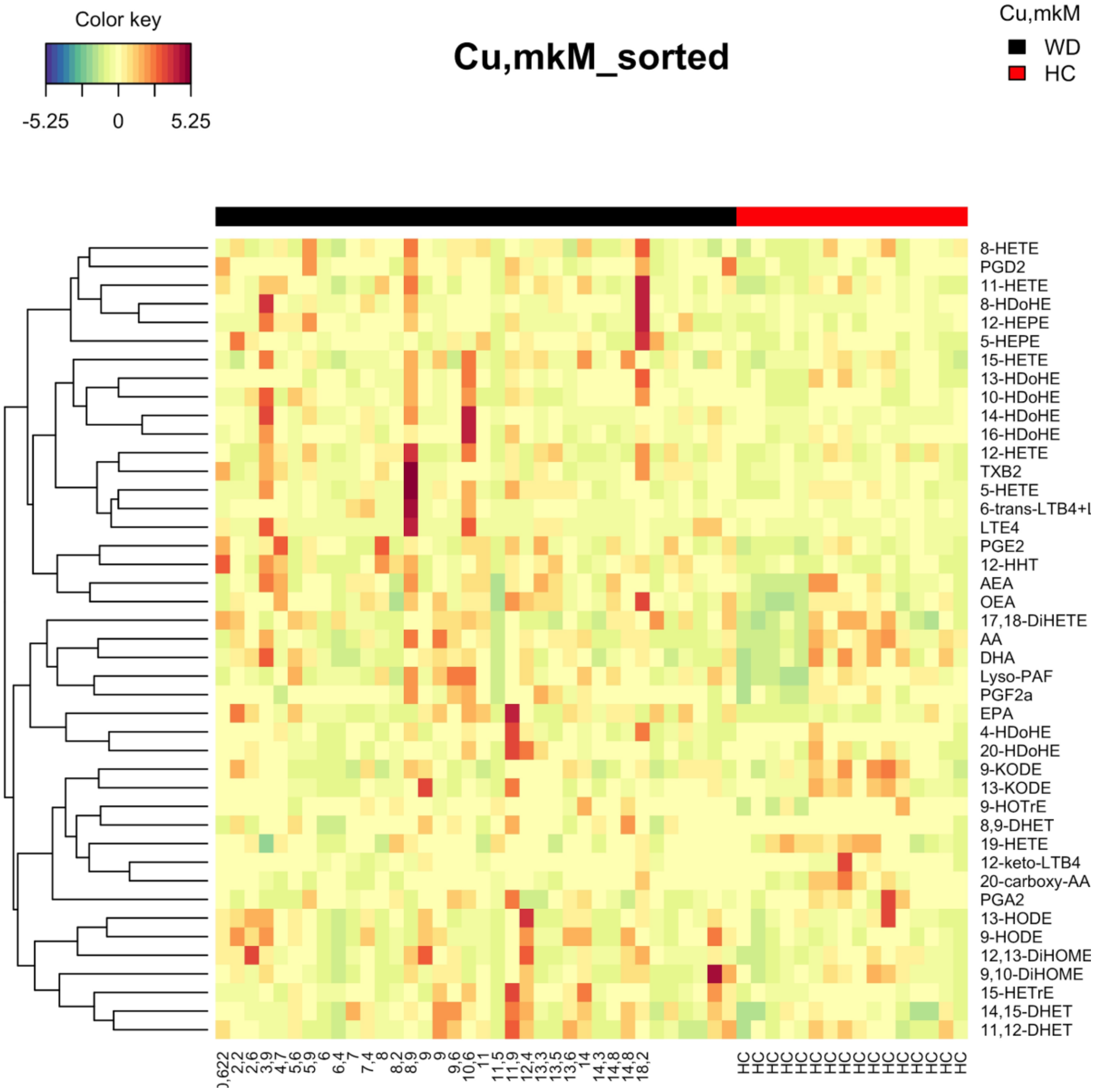
Color key
-5.25
0
5.25
Cu,mkM_sorted
Cu,mkM
WD
HC
8-HETE
PGD2
11-HETE
8-HDoHE
12-HEPE
5-HEPE
15-HETE
13-HDoHE
10-HDoHE
14-HDoHE
16-HDoHE
12-HETE
TXB2
5-HETE
6-trans-LTB4+l
LTE4
PGE2
12-HHT
AEA
OEA
17,18-DiHETE
AA
DHA
Lyso-PAF
PGF2a
EPA
4-HDoHE
20-HDoHE
9-KODE
13-KODE
9-HOTrE
8,9-DHET
19-HETE
12-keto-LTB4
20-carboxy-AA
PGA2
13-HODE
9-HODE
12,13-DiHOME
9,10-DiHOME
15-HETrE
14,15-DHET
11,12-DHET

B

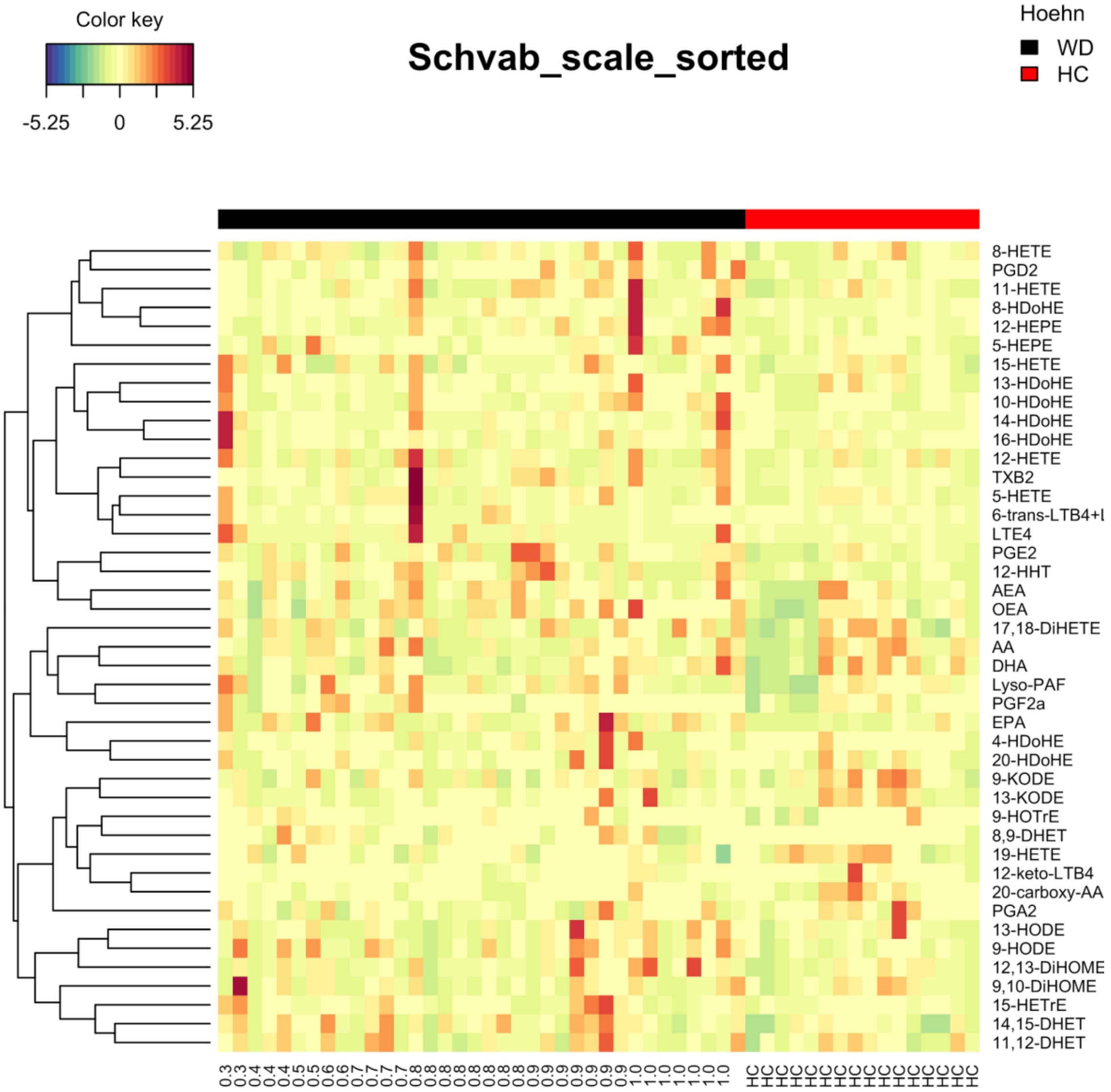
Color key
-5.25
0
5.25
Schvab_scale_sorted
Hoehn
WD
HC
8-HETE
PGD2
11-HETE
8-HDoHE
12-HEPE
5-HEPE
15-HETE
13-HDoHE
10-HDoHE
14-HDoHE
16-HDoHE
12-HETE
TXB2
5-HETE
6-trans-LTB4+l
LTE4
PGE2
12-HHT
AEA
OEA
17,18-DiHETE
AA
DHA
Lyso-PAF
PGF2a
EPA
4-HDoHE
20-HDoHE
9-KODE
13-KODE
9-HOTrE
8,9-DHET
19-HETE
12-keto-LTB4
20-carboxy-AA
PGA2
13-HODE
9-HODE
12,13-DiHOME
9,10-DiHOME
15-HETrE
14,15-DHET
11,12-DHET

C

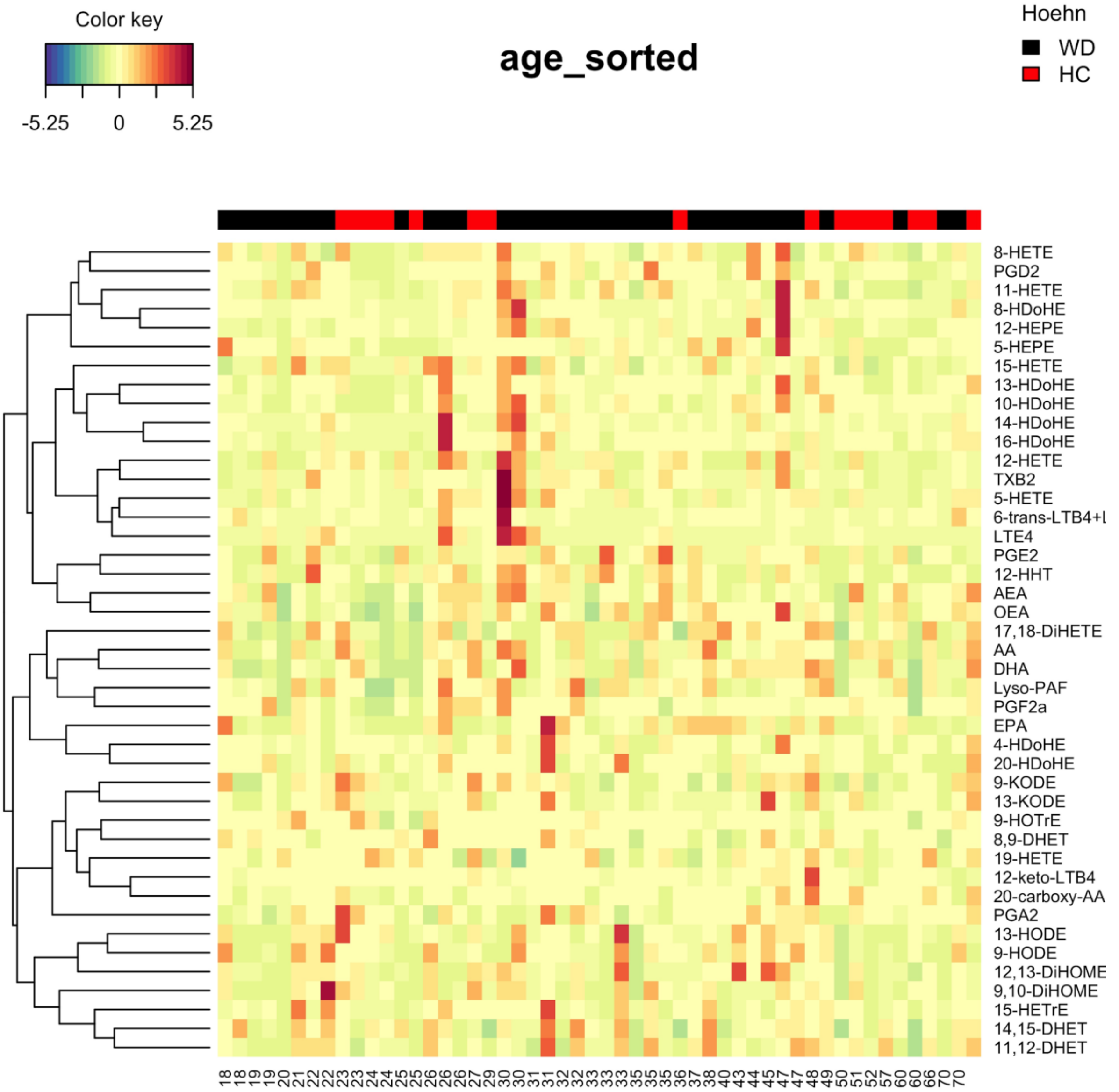
Color key
-5.25
0
5.25
age_sorted
Hoehn
WD
HC
8-HETE
PGD2
11-HETE
8-HDoHE
12-HEPE
5-HEPE
15-HETE
13-HDoHE
10-HDoHE
14-HDoHE
16-HDoHE
12-HETE
TXB2
5-HETE
6-trans-LTB4+l
LTE4
PGE2
12-HHT
AEA
OEA
17,18-DiHETE
AA
DHA
Lyso-PAF
PGF2a
EPA
4-HDoHE
20-HDoHE
9-KODE
13-KODE
9-HOTrE
8,9-DHET
19-HETE
12-keto-LTB4
20-carboxy-AA
PGA2
13-HODE
9-HODE
12,13-DiHOME
9,10-DiHOME
15-HETrE
14,15-DHET
11,12-DHET

D

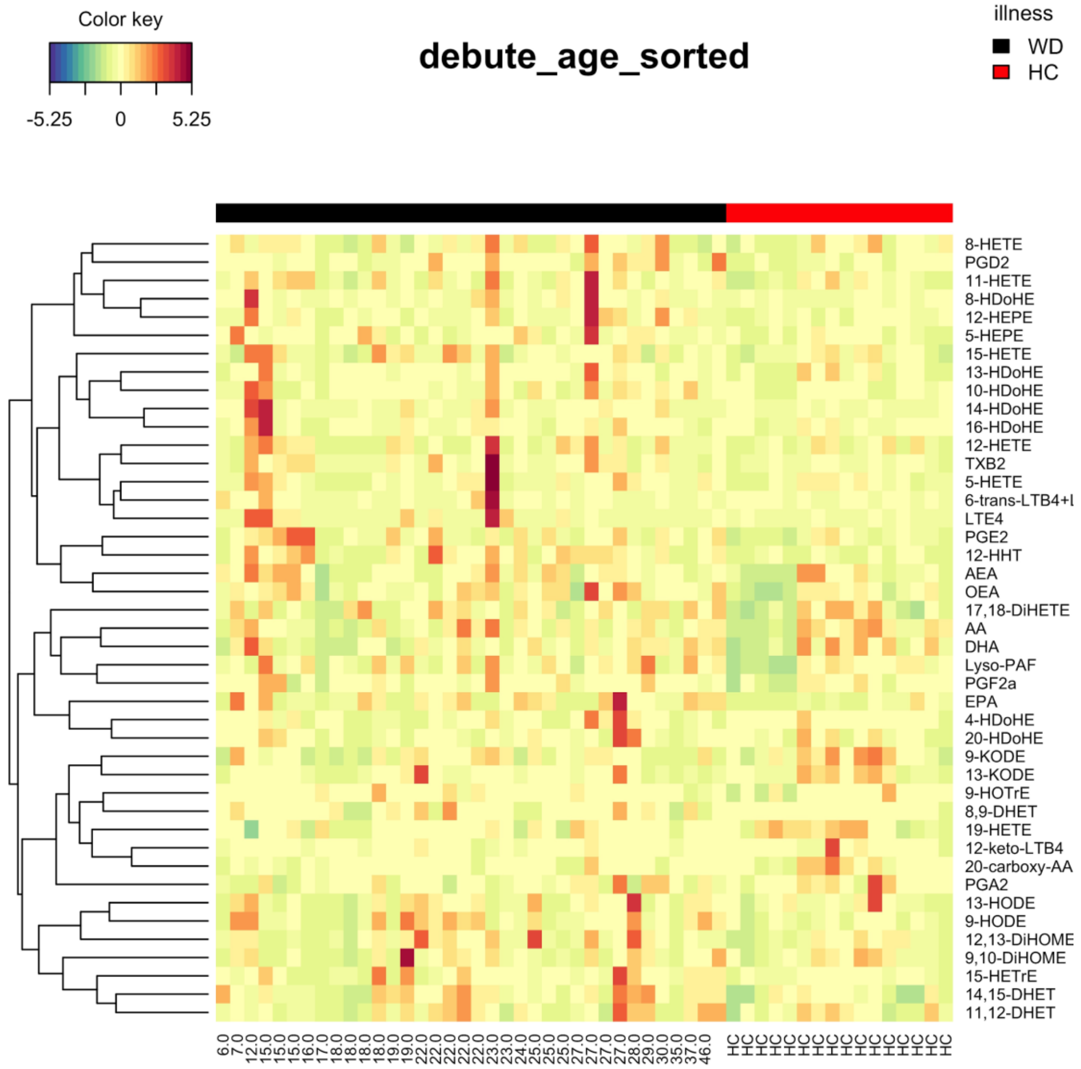

Figure S3 (A-D). A clustered image map was generated using euclidean distance and complete linkage clustering algorithm. On the figure each entry of that matrix is colored according to its value, rows represent subjects, columns - metabolites. Dendrograms are shown on the left side (for metabolites). Clustering was performed only for rows, columns are placed in ascending order according to studied characteristic (A) Cu, mkM in serum, (B) Shvab scale, (C) age, (D) debut age. Studied parameters values are labeled on the bottom side of CIM. Colorbar on the top side of the picture indicates whether the subject is WD (black) or HC (red).

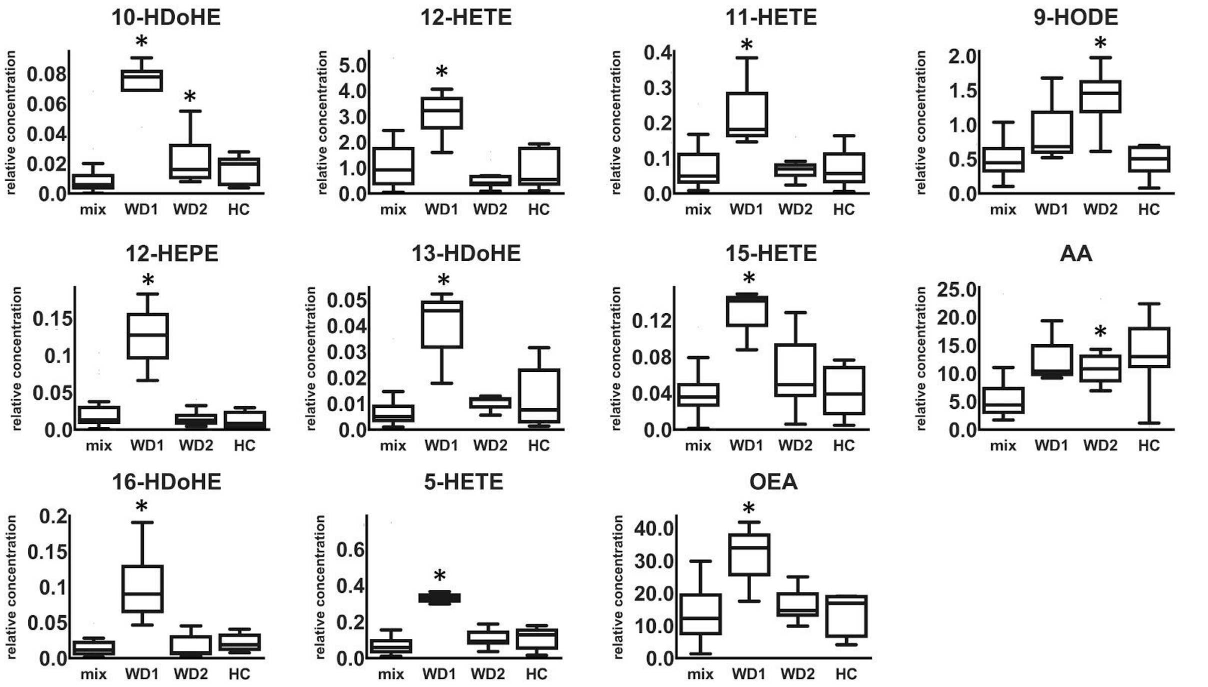

Figure S4. Relative concentrations of separate metabolites which changed significantly in WD1 and/or WD2 in comparison with HC. Pairwise comparison of adjusted means was conducted taking into account age and sex of patients. * - p<0.05 (adjusted for multiple testing).

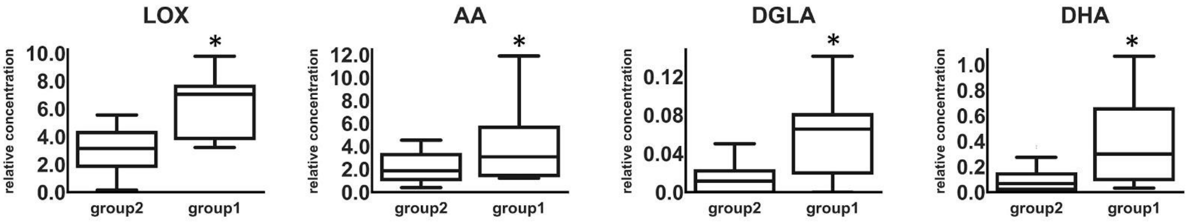

Figure S5. Relative concentrations of summed metabolites which changed significantly between group1 and group2. Concentrations of compounds were summed according to their acid precursors (AA, DHA, EPA, ALA, DGLA, EA, EPA) or via which metabolic pathways they were derived (COX, CYP, LOX or non-enzymatic ROS). Here only compounds which significantly differs between groups are shown. Pairwise comparison of adjusted means was conducted taking into account age and sex of patients. * - p<0.05 (adjusted for multiple testing).
